# Pituitary Apoplexy Secondary to Gonadotropin-Releasing Hormone Agonist for Breast Cancer

**DOI:** 10.1210/jcemcr/luad069

**Published:** 2023-06-28

**Authors:** Katherine Mastandrea, Mihail Voica, Maryam Tetlay, Farhad Hasan

**Affiliations:** Division of Endocrinology, Department of Medicine, Allegheny General Hospital, Pittsburgh, PA 15090, USA; Division of Endocrinology, Department of Medicine, Allegheny General Hospital, Pittsburgh, PA 15090, USA; Division of Endocrinology, Department of Medicine, Allegheny General Hospital, Pittsburgh, PA 15090, USA; Division of Endocrinology, Department of Medicine, Allegheny General Hospital, Pittsburgh, PA 15090, USA

**Keywords:** pituitary apoplexy, GnRH agonist, breast cancer

## Abstract

Pituitary apoplexy is a potentially fatal clinical condition that results from pituitary infarction due to ischemia or hemorrhage. We present a case of a 53-year-old female patient with a history of recurrent pituitary macroadenoma who presented with headache, blurry vision, nausea, vomiting, and photophobia after receiving a gonadotropin-releasing hormone (GnRH) agonist, leuprolide, as part of adjuvant endocrine therapy for breast cancer. Magnetic resonance imaging (MRI) confirmed the presence of pituitary apoplexy, and endocrine workup showed anterior hypopituitarism. The patient was managed conservatively and required glucocorticoid replacement. A repeat MRI after 3 months showed a partially empty sella. A review of the literature revealed that this case adds to the growing number of patients who present with headache, visual symptoms, and symptoms related to meningeal irritation after administration of GnRH agonists, with varying latency from treatment to symptom onset. Although there are other cases involving female patients or patients with known pituitary macroadenomas, to our knowledge, this is the first reported case of pituitary apoplexy in a patient receiving a GnRH agonist as an adjuvant for breast cancer. Physicians should be aware of this rare complication of GnRH agonist therapy in patients with a pituitary macroadenoma.

## Introduction

Pituitary apoplexy is a rare clinical syndrome that occurs when there is ischemia or hemorrhage of the pituitary. It causes a sudden onset of symptoms, which may include headache, visual field deficits, altered mental status, nausea, and vomiting. Most cases of pituitary apoplexy appear in the setting of a pituitary macroadenoma, occurring in 2% to 12% of known pituitary adenomas [1]. Most cases occur spontaneously, but predisposing factors include pregnancy, diabetes, hypertension, head trauma, and most pertinent to this case, treatment with gonadotropin-releasing hormone (GnRH) agonists. The majority of these (>75%) are men undergoing treatment for prostate cancer, but our literature search has also yielded 5 cases of women presenting with pituitary apoplexy who were receiving GnRH agonists for different indications: oocyte donation, in vitro fertilization, fibroids, and GnRH dynamic diagnostic testing (Table 1). GnRH agonists can be used, in addition to aromatase inhibitors, to deplete estrogen in perimenopausal women with hormone-sensitive disease. Our patient presented with pituitary apoplexy following the administration of leuprolide as an adjuvant therapy for breast cancer.

**Table 1.** Published cases of pituitary apoplexy following treatment with GnRH agonists in female patients

First author. Year	Study type	Known pituitary adenoma	Patient characteristics		Indication for treatment	Medication and dose	Following GnRH agonist administration		Symptoms	Subsequent hormone replacement
			Age (y)	Gender			Onset of apoplexy	Dose numbers		
*Arafah, B* [2]. *1989*	*Case Report*	*Yes*	*41*	*Female*	*Pituitary adenoma*	*GnRH 100 micrograms*	*60 minutes*	*1*	*HA, N/V, VFD, F*	*None*
Engel, G [3]. 2003	Case Report	No	22	Female	Ovum donation	leuprolide, daily	3 days	3	HA, N/V, F, neck stiffness	Thyroid, Glucocorticoids
Jaggi, S [4]. 2019	Case Report	No	32	Female	Infertility	leuprolide, dose ND	30 minutes	1	HA, N/V	None
Triviño, V [5]. 2019	Case Report	No	46	Female	Menorrhagia	triptorelin	5 minutes	1	HA, N/V, VFD	Not Reported
Stefaniak, A [6]. 2020	Case Report	No	33	Female	IVF	triptorelin 0.1 mg/day	12 days	5	HA, N/V, F, neck stiffness	Thyroid, Glucocorticoids
Our Patient, 2020	Case Report	Yes	52	Female	Breast CA	leuprolide 11.25 mg	4 days	1	HA, N/V, PHOTO, VFD	Thyroid, Glucocorticoids

Italicized row is a case of a female individual undergoing dynamic testing of the anterior pituitary function because she had a pituitary adenoma, one of the earliest case reports we found that associated GnRH administration with pituitary apoplexy. Abbreviations: F, fever; GnRH, gonadotrophin-releasing hormone; HA, headache; IVF, in vitro fertilization; N, nausea; NA, not applicable; OP, ophthalmoplegia; PHOTO, photophobia; V, vomiting; VFD, visual field deficit.

## Case Presentation

**Figure 1.**
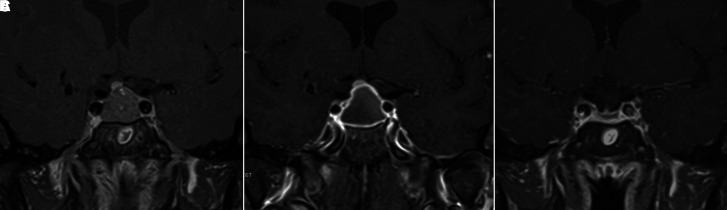
Gadolinium-enhanced pituitary MRI (T1 coronal views). Initial MRI showed 1.5 cm × 1.5 cm × 1.5 cm recurrent pituitary macroadenoma (A). The patient was subsequently diagnosed with breast cancer and given a GnRH agonist for treatment. She presented 4 days later with headache, nausea, vomiting, photophobia, and visual field deficit. MRI during hospitalization showed poor contrast enhancement within the lesion (B), concerning for intratumoral hemorrhage. She was treated conservatively, and a 3-month follow-up MRI showed a partially empty sella (C).

A 53-year-old female patient was initially evaluated in the endocrinology office for a recurrent pituitary macroadenoma. She was originally diagnosed at the age of 37, during a workup for chronic headaches. Evaluation showed a nonfunctioning pituitary macroadenoma with compressive symptoms. She underwent transsphenoidal surgery, after which she was lost to follow-up. She presented to our clinic at the age of 52 for worsening headaches and fatigue. A pituitary magnetic resonance imaging (MRI) scan (Fig. 1A) showed recurrence of the pituitary macroadenoma, measuring 1.5 cm × 1.5 cm × 1.5 cm with abutment of the optic chiasm. The examination was remarkable for obesity (body mass index 34 kg/m^2^) and no visual field defects. Endocrine evaluation showed anterior pituitary hormones within reference ranges, and normal AM serum cortisol (Table 2). However, her serum free thyroxine (T4) was low at 0.7 ng/dL (0.8-1.8 ng/dL) [9.01 pmol/L]. She was started on levothyroxine 50 mcg daily for central hypothyroidism, and tumor surveillance was planned in 6 months.

**Table 2.** Pre- and post-apoplexy plasma hormone concentrations in our patient

Hormone tested	Pre-apoplexy	Post-apoplexy	Normal range
Cortisol	14.2** **mcg/dL (391.8 nmol/L)	**4 mcg/dL** **(110.4 nmol/L)**	6-18.4** **mcg/dL (165.5-507.6 nmol/L)
ACTH	23 pg/mL (5.1 pmol/L)	**3 pg/mL** **(0.6 pmol/L)**	10-48 pg/mL (2.2-10.56 pmol/L)
LH	1.2** **mIU/m (1.2 IU/L)	**<1 mIU/mL** **(<IU/L)**	1-11** **mIU/mL (1-11 IU/L)
FSH	6.5** **mIU/mL (6.5 IU/L)	**1.4 mIU/mL** **(1.4 IU/L)**	2.5-9.1** **mIU/mL (2.5-9.1 IU/L)
IGF-1	101 ng/mL (13.23 nmol/L)	**52 ng/mL** **(6.81 nmol/L)**	70-215 ng/mL (9.2-28.2 nmol/L)
Prolactin	10.1 ng/mL (10.1 ng/mL)	**2.3 ng/mL** **(2.3 ng/mL)**	3-18.6 ng/mL (3-18.6 ng/mL)
TSH	2.5** **mIU/L (2.5 IU/L)	0.9** **mIU/L (0.9 IU/L)	0.4-4.5** **mIU (0.4-4.5 IU/L)
Free T4	**0.7 ng/dL** **(9 pmol/L)**	0.9 ng/dL (11.6 pmol/L)	0.8-1.8 ng/dL (10.3-23.2 pmol/L)

Abnormal values are shown in bold font. Values in parenthesis are International System of Units (SI). Abbreviations: ACTH, adrenocorticotropic hormone; FSH, follicle-stimulating hormone; IGF-1, insulin-like growth factor 1; LH, luteinizing hormone; T4, thyroxine; TSH, thyrotropin (thyroid-stimulating hormone).

She was subsequently diagnosed with stage T3N2M0, estrogen receptor/progesterone receptor (ER/PR)-positive invasive lobular carcinoma of the left breast. She underwent a partial mastectomy with adjuvant chemotherapy and radiation. Given that she was perimenopausal with hormone-sensitive, metastatic disease, endocrine therapy was planned to follow with anastrozole and leuprolide. She received her first dose of intramuscular leuprolide 11.25 mg on June 9, and she presented to the emergency room on June 12 (4 days later) with acute onset severe headache with associated photophobia, phonophobia, nausea, emesis, and blurry vision in her right eye.

## Diagnostic Assessment

Physical examination revealed a difference in pupil size, with right pupil 2 mm and left pupil 4 mm, with both equally reactive. Ophthalmologic evaluation showed mild bilateral impairment in visual acuity, (20/30), and scotomas in the right eye on visual field testing. Fundoscopy revealed advanced cupping of the optic nerve head, consistent with advanced glaucoma, no edema. Given the onset of symptoms after GnRH agonist administration and known pituitary macroadenoma, pituitary apoplexy was suspected. MRI was performed (Fig. 1B), which confirmed the presence of pituitary apoplexy. Subsequent endocrine workup revealed anterior hypopituitarism. Laboratory specimens were drawn prior to intravenous (IV) glucocorticoid therapy and showed anterior hypopituitarism (Table 2). Of note, her serum free T4 was normal, but she was already on thyroid supplementation.

## Treatment

Given that her fundoscopic exam was more consistent with a severe glaucoma, without acute visual field loss or cranial neuropathy, there was low suspicion for the apoplexy affecting the optic chiasm or cavernous sinus. Therefore, conservative treatment was elected, and the patient did not undergo neurosurgical intervention. She experienced improvement of her symptoms following administration of glucocorticoids and was ultimately discharged on hydrocortisone 15 mg/day and continued her levothyroxine 50 mcg daily.

## Outcome and Follow-up

The patient continued to follow up with both neurosurgery and endocrinology as an outpatient. A repeat pituitary MRI in 3 months showed a partial empty sella (Fig. 1C). The patient has been continued on a regimen of hydrocortisone 10 mg in the morning, 5 mg in the afternoon for her central adrenal insufficiency. Her levothyroxine dose is currently 75 mcg, now titrated based on the serum T4 concentration, given concern for central hypothyroidism after her apoplexy. She followed up with a glaucoma specialist after discharge for her visual symptoms, and a trabeculectomy was performed a few months after discharge. Visual fields remained intact.

## Discussion

We present a novel case of pituitary apoplexy following the administration of the GnRH agonist, leuprolide, as adjuvant for breast cancer treatment in a perimenopausal female. Using the PubMed search engine with entry of a combination of key words “*GnRH agonist* and *pituitary apoplexy*” we identified a total of 29 cases of pituitary apoplexy after GnRH agonist administration in the literature. Of these, 24 were cases of pituitary apoplexy in male patients who received GnRH agonists for prostate cancer or while undergoing dynamic pituitary diagnostic testing with GnRH agonists. In this review, we focus on the 5 reported cases of pituitary apoplexy in female patients, who were receiving GnRH agonists for menorrhagia, ovum donation, dynamic pituitary diagnostic testing, and 2 who were receiving it for fertility (Table 1). To our knowledge, this is the first reported case of pituitary apoplexy in a patient receiving a GnRH agonist as an adjuvant for breast cancer.

Our patient's case was unique not only in the indication for her treatment, but also because she had a known pituitary adenoma, which had recurred after having a transsphenoidal resection 15 years prior. She was lost to follow-up after this surgery, and soon after re-establishing with endocrine providers, was unfortunately diagnosed with breast cancer that had spread to regional lymph nodes. Her treatment course included both cancer resection, as well as systemic therapy. Systemic therapy included using ovarian suppression in the form of a GnRH agonist, in addition to aromatase inhibitors, to prevent the spread of disease. These have been shown in the literature to increase the risk for apoplexy in patients with known pituitary adenomas. Our literature search yielded 6 other cases where a patient with a known pituitary adenoma was administered a GnRH agonist and experienced pituitary apoplexy. One of these cases was in a female individual undergoing dynamic testing of the anterior pituitary function because she had a pituitary adenoma, and this was one of the earliest case reports we found that associated GnRH administration with pituitary apoplexy (Table 1, *italicized*). In this case, there was a likely consideration of the benefits of this therapy, and pituitary apoplexy was not recognized yet as a possible outcome.

A review of the 29 case reports showed several symptoms that were present in almost all cases of apoplexy, and these are well represented by the 5 selected cases below (Table 1). Headache, changes in vision, nausea, and emesis were the most common, with signs of meningeal irritation (fever, neck stiffness, photophobia, phonophobia) also being very prevalent. The onset of these symptoms after the administration of GnRH agonist therapy has varied widely, from minutes to several months in some cases. The pathophysiology behind this process is presently unknown, but several hypotheses have been offered to explain this discrepancy. Guerra et al provided a hypothesis that includes acute and subacute pathophysiology [7]. Acutely, the increased secretion of luteinizing hormone (LH), combined with the structural abnormalities of the capillaries supplying the adenoma, causes ischemia in the poorly perfused pituitary tissues. This would correspond with cases in which the patient had symptoms of pituitary apoplexy within minutes to hours of GnRH agonist administration. Other patients, whose presentation was days to months later, could be better explained by the subacute process in which the increase in the size of the tumor causes it to outgrow its blood supply, causing ischemia as well as increased intrasellar pressure. This may represent the process that best fits our patient, who presented 4 days after treatment, as well as several others reviewed in Table 1 below, whose presentation ranged from 3 to 12 days.

The number of case reports involving pituitary apoplexy following GnRH agonist administration has increased significantly in the past 5 to 10 years, likely both because this phenomenon has become more recognized and GnRH agonists continue to have a significant role in a variety of treatment modalities. Our case adds to this number, with breast cancer treatment with GnRH adjuvant therapy posing a risk for pituitary apoplexy. This case also highlights the common clinical features and delayed onset that can be observed, reinforcing that there should be a high index of suspicion for apoplexy in patients who present with headache and visual field changes who are being treated with GnRH agonists.

## Learning Points

- Pituitary apoplexy should be suspected in patients who are being treated with GnRH agonists who present with headache, nausea, vomiting, or visual changes.
- Pituitary apoplexy often occurs acutely after administration of GnRH agonists (minutes to hours), but there are many cases where presentation was weeks to months after treatment.
- Breast cancer is among the conditions that are treated with GnRH agonists, and there is a risk for pituitary apoplexy in patients treated for this indication, especially those with a history of pituitary adenoma.

## Contributors

All authors made individual contributions to authorship. F.H. and M.V. were involved in the diagnosis and management of this patient; M.T. was involved in poster presentation; and K.M. and F.H. were involved in manuscript submission. All authors reviewed and approved the final draft.

## Data Availability

Original data generated and analyzed during this study are included in this published article.

## Abbreviations

GnRH: gonadotrophin-releasing hormone
MRI: magnetic resonance imaging
T4: thyroxine
